# High-quality chest CT segmentation to assess the impact of COVID-19 disease

**DOI:** 10.1007/s11548-021-02466-2

**Published:** 2021-08-06

**Authors:** Michele Bertolini, Alma Brambilla, Samanta Dallasta, Giorgio Colombo

**Affiliations:** Department of Mechanical Engineering, Politecnico di Milano, Milan, Italy

**Keywords:** COVID-19, Segmentation, CT, Respiratory system, Lungs

## Abstract

**Purpose:**

COVID-19 has spread rapidly worldwide since its initial appearance, creating the need for faster diagnostic methods and tools. Due to the high rate of false-negative RT-PCR tests, the role of chest CT examination has been investigated as an auxiliary procedure. The main goal of this work is to establish a well-defined strategy for 3D segmentation of the airways and lungs of COVID-19 positive patients from CT scans, including detected abnormalities. Their identification and the volumetric quantification could allow an easier classification in terms of gravity, extent and progression of the infection. Moreover, these 3D reconstructions can provide a high-impact tool to enhance awareness of the severity of COVID-19 pneumonia.

**Methods:**

Segmentation process was performed utilizing a proprietary software, starting from six different stacks of chest CT images of subjects with and without COVID-19. In this context, a comparison between manual and automatic segmentation methods of the respiratory system was conducted, to assess the potential value of both techniques, in terms of time consumption, required anatomical knowledge and branch detection, in healthy and pathological conditions.

**Results:**

High-quality 3D models were obtained. They can be utilized to assess the impact of the pathology, by volumetrically quantifying the extension of the affected areas. Indeed, based on the obtained reconstructions, an attempted classification for each patient in terms of the severity of the COVID-19 infection has been outlined.

**Conclusions:**

Automatic algorithms allowed for a substantial reduction in segmentation time. However, a great effort was required for the manual identification of COVID-19 CT manifestations. The developed automated procedure succeeded in obtaining sufficiently accurate models of the airways and the lungs of both healthy patients and subjects with confirmed COVID-19, in a reasonable time.

## Introduction

Coronavirus disease 2019 (COVID-19) is a highly infectious disease caused by the severe acute respiratory syndrome coronavirus 2 (SARS-CoV-2). The first human cases of COVID-19 were recognized in Wuhan, Hubei, China, in late December 2019. By metagenomic RNA sequencing and virus isolation from throat swab samples, a novel coronavirus was identified as the causative agent of this emerging disease [1]. SARS-CoV-2 is a beta coronavirus that is constituted by a single-stranded RNA structure belonging to the Coronaviridae family [2]. Typical features of COVID-19 include endothelial barrier disruption, dysfunctional alveolar-capillary oxygen transmission, reduced oxygen diffusion capacity, alveolar wall thickening, increased vascular permeability and pulmonary oedema [3].

The reference standard in COVID-19 diagnosis is the reverse transcription polymerase chain reaction (RT-PCR). However, the sensitivity of RT-PCR tests is not perfect, with the possibility of false-negative test results [4]. Several reports have indicated a possible role for chest computed tomography (CT) examination in presumptive diagnosis in patients with a high suspicion of infections of SARS-CoV-2. CT imaging may also be helpful in monitoring disease evolution and evaluating therapeutic efficacy [4–6].

Utilizing CT images, it is possible to obtain 3D models, by means of segmentation, to allow the assessment of the extent and the distribution of the pathology, providing a useful tool for clinicians. Moreover, a volumetric quantification of the affected regions of the lung lobes can be performed [7]. Actually, both CT and magnetic resonance imaging (MRI) technologies could be adopted for the imaging and measurement of airways; however, CT is the established modality in the field [8].

The medical and bioengineering literature refers to different issues relating to image segmentation for the study of the respiratory system. Besides the quality of scans themselves (e.g. resolution and slice thickness), the appearance of airways on CT scans depends on their diameter and the orientation with respect to the plane of the slice [9]. The largest airways are easier to detect because of their dimensions, the thickness of their walls and the more homogeneous grayscale values [9]. Due to the size of the voxel, thin or stenosed airways can appear broken or discontinuous, resulting in both under- and over-segmentation errors [10]. Moreover, if the airways appear thin or disrupted, the segmentation can erroneously leak, due to the similar intensity and texture of the surroundings [11]. These issues, together with other image artefacts, lead to the impossibility of determining a global threshold in terms of Hounsfield Units (HU) to segment the airways completely. Additionally, the optimal threshold differs for large versus small airways, due to partial volume effects [10].

Airways segmentation can be performed both manually and through semi-automatic methods. Manual segmentation requires the user to delineate the entire structure slice by slice, resulting in a tedious and extremely time-consuming process: a full identification may require several hours of analysis [12]. The variabilities caused by human interaction and time consumption opened the way to automatic methods. Lo et al. [13] presented a review of 15 different techniques for airways segmentation from CT images. Moreover, there are additional studies in which the authors compare their own automatic algorithm with a manual segmentation, taken as a gold standard, resulting in overall detection sensitivity of about 73% for human data [14], through a morphological approach and 69% sensitivity for canine data [15], through a fuzzy logic approach. Focusing on lungs, there are pulmonary disease processes and anatomical conditions, such as incomplete pulmonary fissures, that may alter their appearance [16]. Since the identification of the lungs is based on the large difference in terms of HU between the air and the surrounding tissues, in the case of dense pulmonary or subpleural abnormalities, some areas may not be included in the segmentation [17]; therefore, specialists need to manually edit the results.

Patients suspected of SARS-CoV-2 infection should undergo non-contrast-enhanced chest CT, using a low-radiation-dose protocol, to minimize radiation burden [4]. A number of studies have been published, discussing the most common CT manifestations in patients affected by COVID-19 [18]. Several imaging findings were reported in more than 70% of proven COVID-19 cases, including ground-glass opacities, bilateral abnormalities, lower lobes involvement and vascular enlargement [18]. Other reported chest CT abnormalities with intermediate incidence involve consolidations, linear opacities, septal thickening and/or reticular pattern, crazy-paving pattern, air bronchogram, pleural thickening, halo signs, bronchiectasis, nodules, bronchial wall thickening and reversed halo signs [18]. Less common findings include pleural effusion, lymphadenopathy and pericardial effusion, which typically occur later in the course of the disease [18]. In Table 1, the most common CT imaging features of COVID-19 [6] are described in detail.

**Table 1:** Most common COVID-19 CT imaging findings, as reported in the literature

Ground-glass opacity (GGO)	Hazy areas characterized by increased lung density with preservation of bronchial and vascular margins (Fig. 1a)
Consolidation	Replacement of alveolar air with pathological fluids, cells or tissues which results in an increase in pulmonary parenchymal density with obscuration of vessels and airway walls margins (Fig. 1b)
Reticular pattern	Thickening of pulmonary interstitial structures (interlobular septa and intralobular lines) which appears like a high number of small linear opacities on CT images (Fig. 1c)
Crazy-paving pattern	Superimposition of reticular pattern on a GGO background, with a characteristic appearance of irregular paving stones (Fig. 1d)
Air bronchogram	Low attenuation bronchi filled with air or gelatinous mucus on a background given by opaque airless lung region
Airway changes	Bronchiectasis and bronchial wall thickening related to the destruction of the bronchial wall, proliferation of fibrous tissue and bronchiectasis
Pleural changes	Pleural thickening and pleural effusion
Fibrosis	Substitution of the cellular component with fibrous tissue stripes (Fig. 1e) during the healing of pulmonary chronic inflammation
Vascular enlargement	Dilation of pulmonary vessels associated with the damage and swelling of the capillary wall caused by the inflammatory agents
Nodule	Rounded or irregular opacity with a diameter lower than 3 cm which is typically associated with viral pneumonia
Halo sign	Nodules surrounded by a ground-glass opacity (Fig. 1f)
Reversed halo sign	Rounded GGO surrounded by a circular consolidation

The Radiological Society of North America (RSNA) published guidelines to reduce the uncertainty and the variability in reporting imaging findings potentially related to COVID-19 [19], identifying four different categories. It is worth noting that CT imaging manifestations discussed above are non-specific and may overlap with other diseases, including other viral pneumonia [4, 18]. Consequently, the diagnostic value of chest CT imaging for COVID-19 is limited and a negative CT examination result does not exclude COVID-19. The diagnostic accuracy of chest CT depends on reader experience and the adopted criteria. Moreover, due to the intrinsic bidimensional nature of CT findings, their interpretation may be quite tricky, possibly precluding the accurate measurements of dimensions such as area or volume.

Given these constraints, the aims of the study were (1) to implement a method for 3D segmentation from CT scans, thereby demonstrating the visible pathological effects induced by COVID-19; (2) to obtain accurate 3D models that can be utilized to assess the impact of the pathology, by volumetrically quantifying the extension into the affected areas; (3) to assist with the interpretation and communication of the CT findings.

In our study, the most appropriate segmentation techniques were evaluated, beginning with manual segmentation. An automatic procedure was then tested on the same reference patients, to assess its effectiveness. After the identification of the potential value of this automatic method, it was utilized for the segmentation of the respiratory system of COVID-19 positive patients. A conclusive comprehensive comparison, to identify the pros and cons of both the methodologies, in terms of time consumption, required anatomical knowledge, and branch detection sensitivity was conducted.

## Materials and methods

The segmentation process was performed using proprietary Mimics software (Materialise, Leuven, Belgium, version 23), starting from different stacks of chest CT images of subjects with and without COVID-19 positive diagnosis. The characteristics of the patients and of the sets of images are listed in Table 2. CT images were downloaded from available online databases [20, 21].

**Table 2:** CT images properties of the patients involved in the study. Patients from 3 to 6 had a COVID-19 positive diagnosis

Patient	Pathology	Age	Sex	Slice Thickness [mm]	N° slices	Resolution [pixel]	Pixel size [mm]
1	Normal	n/a	n/a	0.500	456	512 × 512	0.729
2	Normal	n/a	n/a	0.500	382	512 × 512	0.782
3	COVID-19	70	M	1.500	205	512 × 512	0.633
4	COVID-19	84	F	0.625	510	512 × 512	0.703
5	COVID-19	33	F	0.625	451	512 × 512	0.703
6	COVID-19	60	M	1.500	244	512 × 512	0.740

**Fig. 1:**
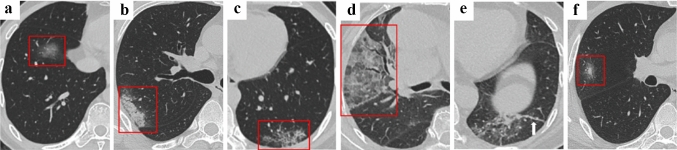
CT images of most common COVID-19 CT findings. Adapted from [6]. **a** Ground-glass opacity. **b** Consolidation. **c** Reticular pattern. **d** Crazy-paving pattern. **e** Fibrosis. **f** Halo sign

The segmentation process of the CT stacks of healthy subjects was carried out manually, to obtain reference segmentation models; then, the automatic *Segment Airway* tool provided by *Mimics Pulmonary Module* was applied on them, to verify its efficacy in airway branches identification. In the case of COVID-19 positive patients, just the automatic *Segment Airway* and *Segment Lungs* tools were utilized. Even if not strictly important for the visualization or quantification of COVID-19 manifestations, lower airways were anyway always included in the models, both to guarantee a more comprehensive comparison between manual and automatic segmentation tools and to provide a clearer and intuitive anatomical representation.

### Healthy subjects segmentation

In the manual segmentation process, a region growing algorithm was used for airway reconstruction, placing a first seed point in correspondence of the trachea. Additional seed points were then placed on the last detected airway branches. This was followed by meticulous manual editing on the single slices. Finally, a global smoothing (Laplacian, I order, with shrinking compensation) and a wrap operation were performed on the converted parts, to enhance the surface finishing and filter small inclusions.

The lungs were segmented first using a thresholding algorithm (threshold − 1024 ÷ − 500 HU); a Boolean operation was used to include just the lung parenchyma, splitting the right and left lung masks. Pulmonary internal vasculature was not included in the segmentation, resulting in masks characterized by vascular cavities; all the isolated vessel branches were then added through combined Boolean operations, to improve the appearance of the final models. The obtained masks were manually edited and then globally smoothed once converted into parts. On the other hand, the automatic Mimics *Segment Airway* tool allowed to segment the airways starting from two seed points, indicating the start of the trachea and its direction. A middle detection level was set to balance branches identification and leakage occurrence. Global smoothing algorithm was applied as in the previous case. The *Mimics Pulmonary Module* additionally provides the centreline-labelling tool, which allows an automatic computation of the number of airway branches.

### COVID-19 positive patient segmentation

In the automatic segmentation process applied on COVID-19 positive patients, the *Segment Airway* tool was exploited to obtain airways 3D models and the centreline was extracted as previously explained. The centreline was needed to automatically segment the lungs through the *Segment Lungs* tool, adopting the default thresholds (− 1024 ÷ − 500 HU). Due to the presence of numerous COVID-19 manifestations, several regions of the lung parenchyma were not included in the masks, because of their high-intensity level. All the masks were refined through a rigorous manual editing operation, to incorporate opacities and correct all the boundaries. Pulmonary vasculature correction and global smoothing were then performed as previously described.

COVID-19 manifestations were identified through accurate visual inspection; a thresholding algorithm was adopted for the segmentation, properly selecting the boundary thresholds case by case and limiting the region of interest (ROI). Afterwards, the obtained masks were filled with one or two voxels levels, to get less sharp contours. The resulting masks were intersected with the masks of the lungs, and then, they were manually adjusted. The final smoothing was limited in this case, to maintain the irregular appearance of the CT findings.

### Post-processing

**Fig. 2:**
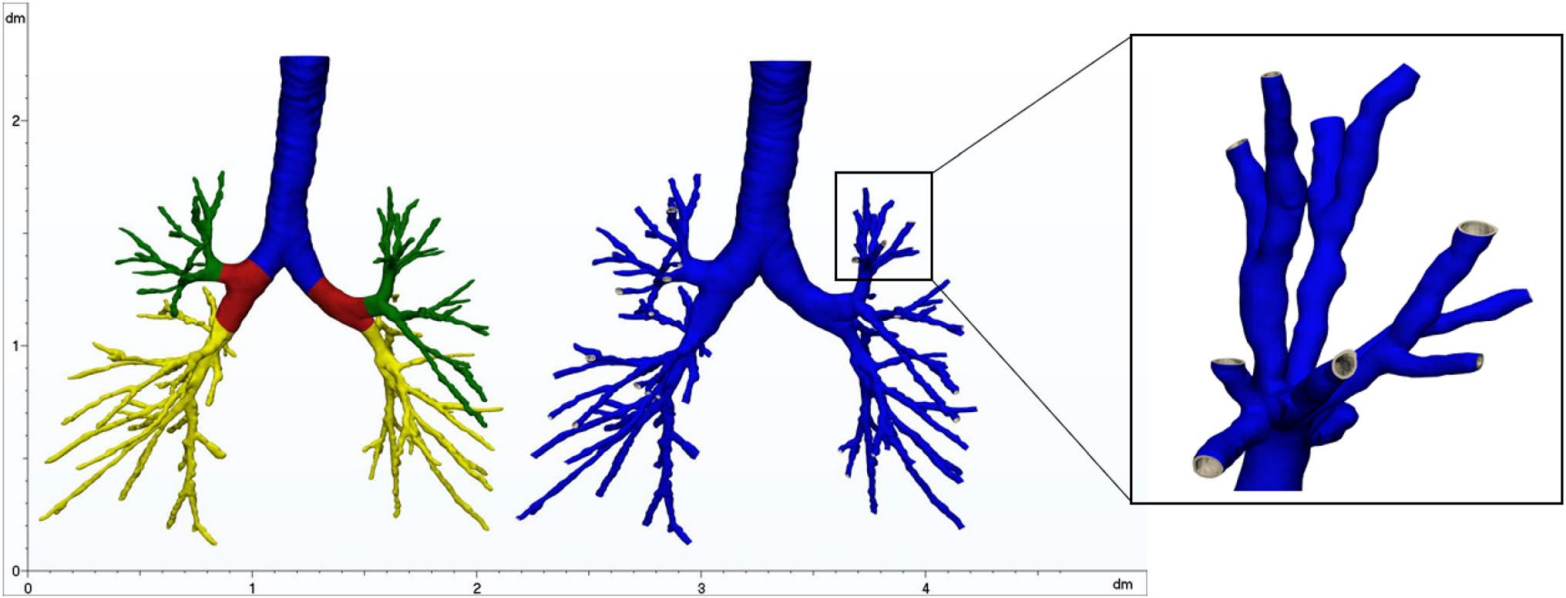
Subdivision of the airways 3D model into different portions, to assign different thickness values (left), and resulting hollowed 3D model (right), in case of Patient 1

All the parts were later imported in 3-Matic software (Materialise, Leuven, Belgium, version 15), and common operations were carried out on all the models. The airways were divided into seven different portions and hollowed towards the outside independently (Fig. 2), assigning different thickness values according to the literature [22]. The different regions were then merged through a Boolean operation, the connecting edges were smoothed properly and all the ends of the branches were opened.

**Fig. 3:**
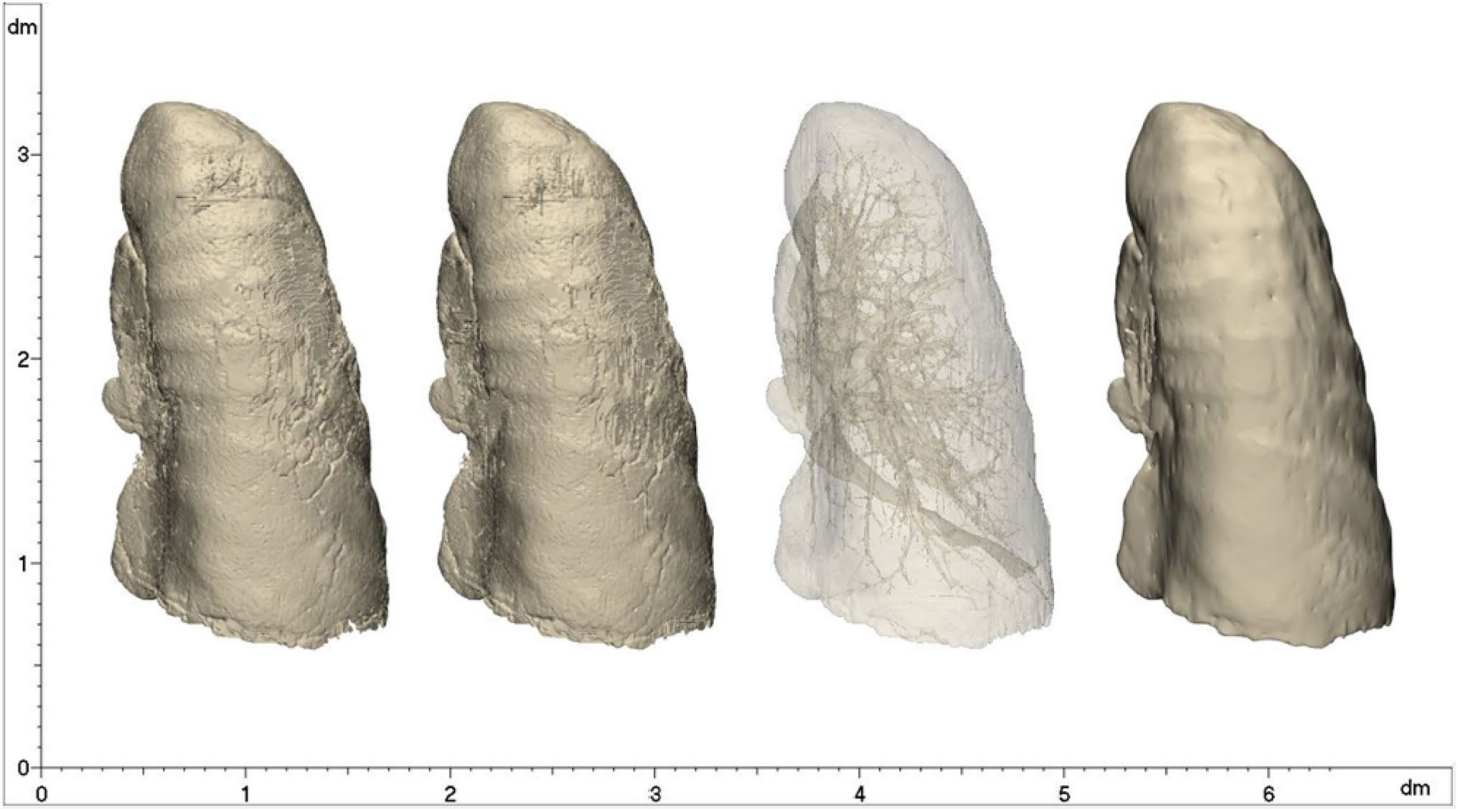
Sequence of the process for the right lung 3D reconstruction in Patient 4: starting from the mask obtained by the automatic method, the contours were manually adjusted; then, the pulmonary vasculature was corrected, and finally, the model was locally and globally smoothed

The local smoothing tool was applied with a proper smoothing strength, to further improve the finishing of the 3D models. The entire process of lung 3D model generation is depicted in Fig. 3, in the case of Patient 4.

At the end of these procedures, manual and automatic segmentation results were compared, in terms of time consumption and reconstruction accuracy and by extracting the number of identified airway branches. (This last comparison was limited to healthy subjects.) Moreover, the volume of both the left and the right lungs was acquired, together with the volume of the identified CT manifestations, to evaluate the global impact of the disease, for Patients 3–6. CT findings are classified according to Table 1.

## Results

The required time for the overall manual segmentation process was approximately 9 h for each patient. The extraction of the airways was the most time demanding process (7 h), and adequate anatomy knowledge and experience were needed. The use of the automatic tools allowed a much faster identification of the airways (approximately 5 min), providing a good level of accuracy. The post-processing operations required nearly 1 h, in both cases. Regarding the manual segmentation of the lungs, about 1 h was needed, also including the post-processing operations. The automatic tools did not succeed in giving satisfactory results in COVID-19 positive patients and about 3 h of manual editing were necessary to obtain the final 3D reconstruction of the lungs. The most time-consuming operation, however, was the segmentation of the COVID-19 CT manifestations, with variable time consumption between 5 and 10 h.

**Fig. 4:**
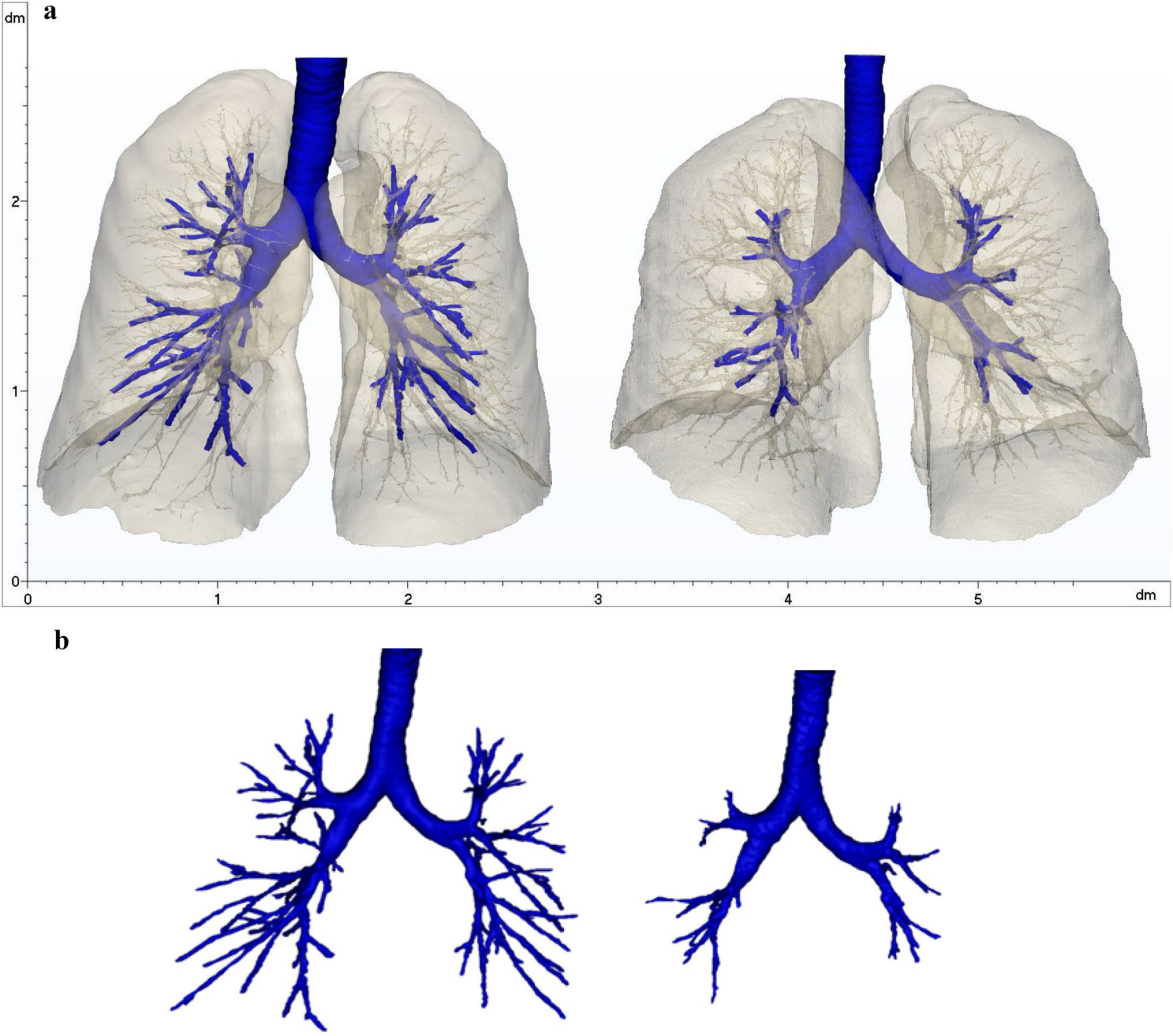
**a** Reconstructed 3D models of the respiratory system of Patient 1 (left) and Patient 2 (right), not affected by COVID-19. **b** Comparison of manual (left) and automatic airways identification results (right) in Patient 1

In Fig. 4a, the final segmentation of the respiratory system of Patient 1 and Patient 2 is reported. A comparison of the manual identification of the airways and automatic algorithm result of Patient 1 is shown in Fig. 4b. The properties of 3D models are listed in Table 3.

**Table 3:** Features extracted from the 3D models of Patients 1 and 2

Patient	Right lung volume [mm^3^]	Left lung volume [mm^3^]	Total volume lungs [mm^3^]	Airway branches (manual)	Airway branches (automatic)
1	2,105,205	1,881,302	3,986,507	175	48
2	2,325,143	2,246,902	4,572,045	87	29

**Fig. 5:**
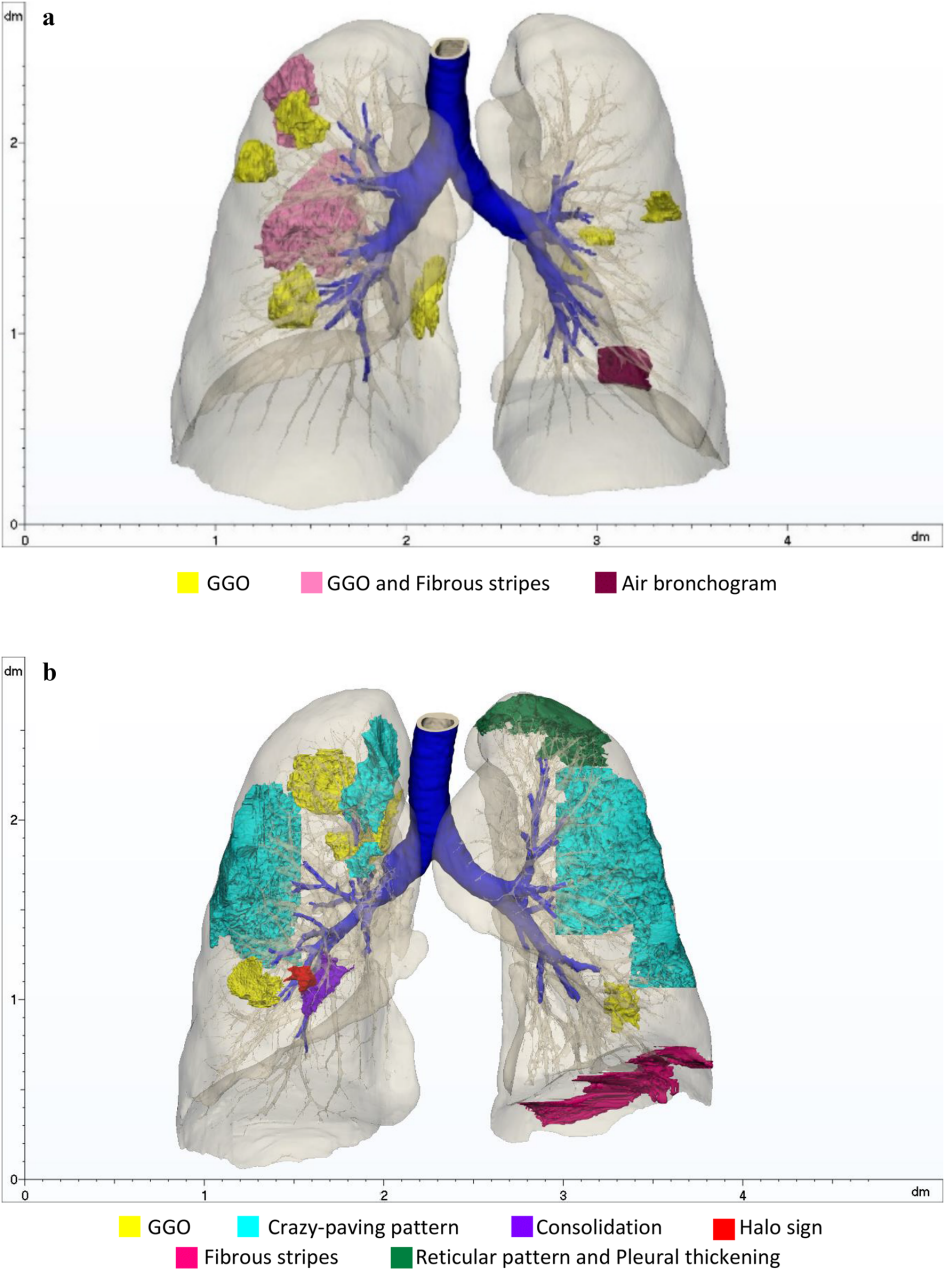

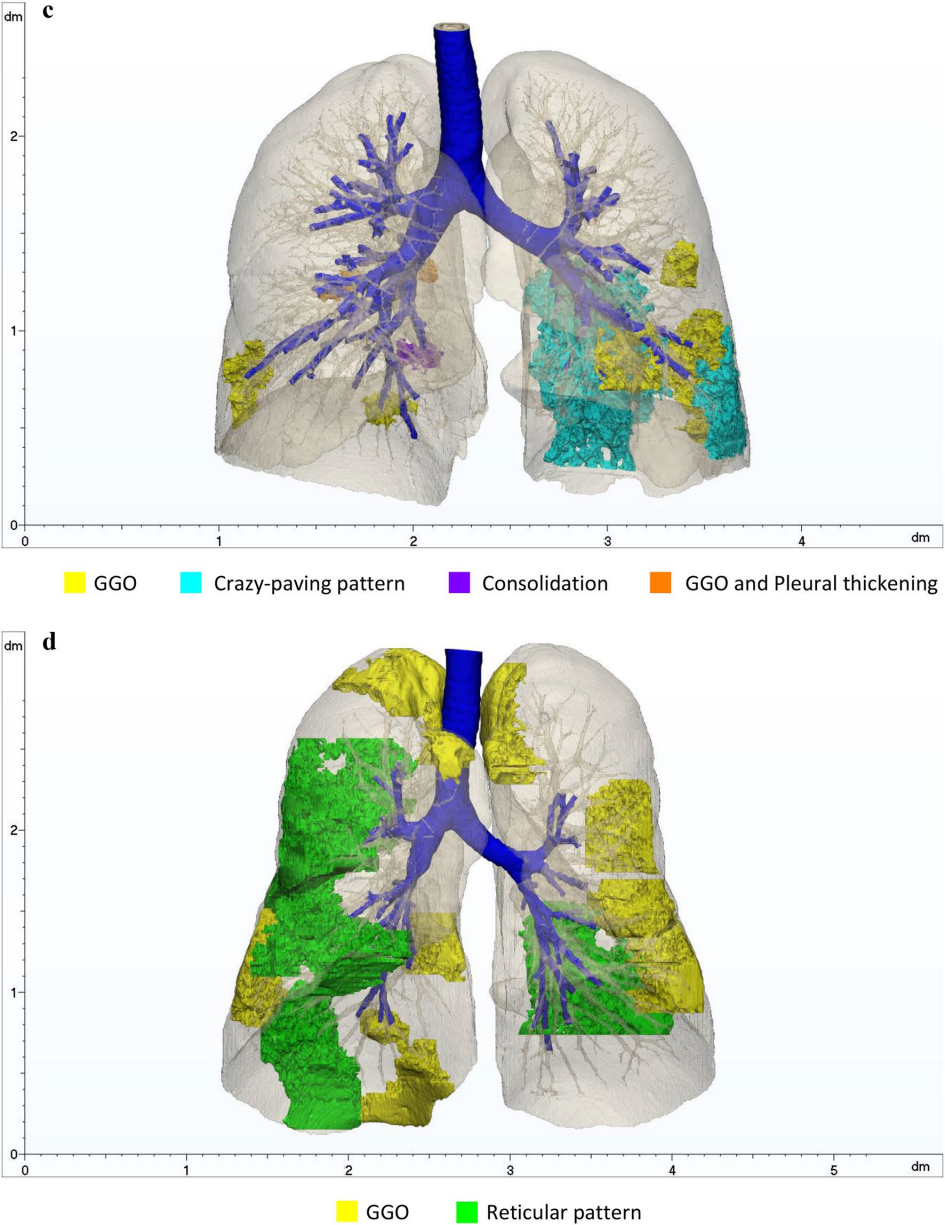
**a** 3D model of the respiratory system of Patient 3 (M, 70 years old, Typical appearance). **b** 3D model of the respiratory system of Patient 4 (F, 84 years old, Typical appearance). **c** 3D model of the respiratory system of Patient 5 (F, 33 years old, Indeterminate appearance). **d** 3D model of the respiratory system of Patient 6 (M, 60 years old, Indeterminate appearance)

In Table 4, the properties of 3D models of patients affected by COVID-19 are listed. In Fig. 5, the final segmentations of the respiratory system of Patients 3 to 6 are reported. Different colours were arbitrarily assigned to the abnormalities, to help visualization.

**Table 4:** Features extracted from the 3D models of Patients 3, 4, 5 and 6

Patient	Right lung volume [mm^3^]	Left lung volume [mm^3^]	Total volume lungs [mm^3^]	Airway branches
3	2,383,213	1,964,480	4,347,693	96
4	2,286,867	1,972,425	4,259,292	93
5	2,106,598	1,808,441	3,915,039	240
6	3,575,102	3,181,443	6,756,545	113

In Table 5, the CT manifestations identified in each case are detailed, together with the Radiological Society of North America (RSNA) classification. The percentage of involvement of the lungs was evaluated, to assess the degree of diffusion of the disease.

**Table 5:** COVID-19 CT manifestations, imaging classification, percentages of the right lung (RL) and left lung (LL) affected by abnormalities and percentage of the extent of the disease computed on the whole lung volume

Patient	CT findings	RSNA classification	Global affected volume [mm^3^]	Affected RL	Affected LL	Global
3	GGO, fibrous stripes, air bronchogram	Typical	97,034	3.68%	0.48%	2.23%
4	GGO, fibrous stripes, crazy-paving pattern, reticular pattern, pleural thickening, halo sign, consolidation	Typical	327,607	6.99%	8.50%	7.69%
5	GGO, crazy-paving pattern, consolidation, pleural thickening	Indeterminate	126,084	0.43%	6.47%	3.22%
6	GGO, reticular pattern	Indeterminate	811,439	15.32%	8.29%	12.01%

Now, a sketched discussion about obtained reconstructions for COVID-19 positive patients is provided, illustrating Table 5 classification.

Patient 3 (male, 70 years old) (Fig. 5; Table 5): Patient 3 presented with predominantly peripheral bilateral GGO formations with rounded morphology, affecting different lung sections. The abnormalities found in the CT scan led to the RSNA classification as *Typical appearance*. Fibrous stripes, typical of the healing phase of the pulmonary chronic inflammation, were also observed close to the pleura. The percentage of the whole lung affected volume suggested a *Moderate* severity of the disease according to the literature [23] and, together with the presence of fibrosis, it may be indicative of a late stage of the viral infection [4, 24].

Patient 4 (female, 84 years old) (Fig. 5; Table 5): Patient 4 displayed signs of rounded GGOs, with the presence of crazy-paving pattern, reticular pattern, halo sign, consolidation, pleural thickening and fibrous stripes, leading to a *Typical appearance* classification. The high percentage of affected lung regions and the typology of CT findings may be representative of a peak stage of infection and of a *Severe* case.

Patient 5 (female, 33 years old) (Fig. 5; Table 5): Patient 5 showed non-rounded GGOs mainly in the left lung, lacking a specific distribution, crazy-paving pattern, consolidation and pleural thickening (*Indeterminate appearance*). Considering the extension of the affected lung region and the CT abnormalities, the infection was classified as *Moderate type* at the peak stage.

Patient 6 (male, 60 years old) (Fig. 5; Table 5): Patient 6 was labelled as *Indeterminate appearance* due to the non-rounded GGOs without a specific distribution and reticular pattern. This was classified as *Severe* disease in a progressive stage.

## Discussion and conclusions

With both manual and automatic methods, it was possible to obtain a realistic reconstruction of the lower respiratory tract and of the pulmonary parenchyma. These 3D models could provide the clinicians with an overall view of the impact of the infection. Furthermore, a volumetric quantification of the CT findings can be performed, helping in the evaluation of the progression of the pathology and of the most proper therapeutic actions. In addition, 3D models provide a powerful tool for communicating the severity of COVID-19.

The use of an automatic algorithm allowed for a substantial reduction in the operating time in the case of airways segmentation; nonetheless, the number of identified branches was lower, in comparison with the detection by the operator through the visual inspection of the CT images. However, the model of the airways obtained with the automatic technique resulted to be more than adequate, if the assessment of COVID-19 impact on the respiratory system is considered the main target. In the case of healthy patients, the manual process of lung segmentation was not particularly time-consuming, nor complex. Whereas, for COVID-19 positive patients, the application of the automatic technique provided unacceptable results, requiring a laborious manual editing operation. Moreover, the greater the severity of the pathology, the worse the automatic segmentation result.

With respect to airway models, it was our observation that the accuracy of the detection was strongly affected by the CT imaging parameters. Comparing Patients 3 and 5, the slice thickness and the number of slices were the most relevant factors to be considered. In agreement with the literature, a low slice thickness determined an enhanced segmentation process [25]. Additionally, it was also observed that a higher number of slices led to a higher number of detected branches. Finally, with worsening levels of disease severity, poor segmentation was noted, despite similar CT scanning parameters, due to lower contrast between the airway walls and the pulmonary parenchyma.

To summarize, the most common COVID-19 CT findings in the patients analysed in this study were the GGOs and, in the case of severe pathology, the affected lung volume was mostly occupied by crazy-paving pattern and reticular pattern. It was also noticed that the infection typically involved the regions near the lung surfaces.

It is important to highlight certain limitations in this study. The present work was aimed at establishing a method to segment the pathological pulmonary manifestations in COVID-19 focusing just on four positive COVID-19 patients. To derive statistically significant data, it would be necessary to analyse a wider population ranging in age. Moreover, due to the lack of information on the clinical history of the analysed patients, it was not possible to avoid potential overlapping of the observed CT manifestations with those of other pathologies.

Anyway, we are confident the segmentation process that has been demonstrated could provide practising radiologists and researchers a tool to visualize, in a composite view, the many different pathological processes that take place in COVID-19 pneumonia, as well as their extent. Chest CTs in patients with active COVID-19 pneumonia are often obtained to assess complications and severity. Their association with 3D segmentation can provide clinicians with a comprehensive “snapshot” of the overall current status of pulmonary involvement that may prove to be useful in patient management. The categories of CT findings in COVID-19 pneumonia have been linked to temporal changes in pathology, from early to late radiological findings. Future studies, analysing the changes in the 3D segmentation images in individual patients over time, may help elucidate, in a more intuitive fashion, the nature of the progression of COVID-19 disease.
